# EGFR/ErbB Inhibition Promotes OPC Maturation up to Axon Engagement by Co-Regulating PIP2 and MBP

**DOI:** 10.3390/cells8080844

**Published:** 2019-08-06

**Authors:** Emanuela Nocita, Alice Del Giovane, Marta Tiberi, Laura Boccuni, Denise Fiorelli, Carola Sposato, Elena Romano, Francesco Basoli, Marcella Trombetta, Alberto Rainer, Enrico Traversa, Antonella Ragnini-Wilson

**Affiliations:** 1 NeurotechIT Laboratory, Department of Biology, University of Rome “Tor Vergata”, 00133 Rome, Italy; 2 Advanced Microscopy Center, Department of Biology, University of Rome “Tor Vergata”, 00133 Rome, Italy; 3 Department of Engineering, Università Campus Bio-Medico di Roma, 00128 Rome, Italy; 4 School of Materials and Energy, University of Electronic Science and Technology of China, Chengdu 611731, China

**Keywords:** remyelination, EGFR inhibitor, smoothened agonist, microfibers, drug screening

## Abstract

Remyelination in the adult brain relies on the reactivation of the Neuronal Precursor Cell (NPC) niche and differentiation into Oligodendrocyte Precursor Cells (OPCs) as well as on OPC maturation into myelinating oligodendrocytes (OLs). These two distinct phases in OL development are defined by transcriptional and morphological changes. How this differentiation program is controlled remains unclear. We used two drugs that stimulate myelin basic protein (MBP) expression (Clobetasol and Gefitinib) alone or combined with epidermal growth factor receptor (EGFR) or Retinoid X Receptor gamma (RXRγ) gene silencing to decode the receptor signaling required for OPC differentiation in myelinating OLs. Electrospun polystyrene (PS) microfibers were used as synthetic axons to study drug efficacy on fiber engagement. We show that EGFR inhibition per se stimulates MBP expression and increases Clobetasol efficacy in OPC differentiation. Consistent with this, Clobetasol and Gefitinib co-treatment, by co-regulating RXRγ, MBP and phosphatidylinositol 4,5-bisphosphate (PIP2) levels, maximizes synthetic axon engagement. Conversely, RXRγ gene silencing reduces the ability of the drugs to promote MBP expression. This work provides a view of how EGFR/ErbB inhibition controls OPC differentiation and indicates the combination of Clobetasol and Gefitinib as a potent remyelination-enhancing treatment.

## 1. Introduction

The myelin sheath insulates axons of the central nervous system (CNS), allowing neural impulses to be transmitted rapidly along axons. Remyelination is the natural process that restores damaged myelin and thereby neuronal function in the adult brain. This ability declines as a consequence of aging or during the progressive phases of multiple sclerosis (MS) [1,2,3]. Pharmacologically-induced remyelination has been envisaged in co-therapies with disease modifying agents for relapsing remitting and secondary progressive MS (RRMS and SPMS, respectively), not only to restore neuronal function, but also for its protective potential on neurodegeneration [1,2,3,4].

A number of bioactive drugs, such as Clobetasol, Gefitinib, Miconazole and Benztropine, have been successfully repositioned by screening FDA-approved compound libraries for their ability to promote myelin basic protein (MBP) expression in cell-based assays that employed either primary murine Oligodendrocyte Precursor Cells (OPCs) [5,6,7], mouse OPC cell lines such as Oli-neuM [8] or Epiblast-derived OPCs (EpiSC-OPC) [9]. However, a rational use of such drugs in remyelination therapies awaits the clarification of their molecular targets [1,2,3,4,5,6,7,8,9,10].

Remyelination essentially relies on two separate developmental steps: Oligodendrogenesis and Oligodendrocyte Precursor Cell (OPC) differentiation into myelinating OLs [1,3]. Promyelinating drugs can act either by promoting oligodendrogenesis and/or by enhancing OPC differentiation into mature OLs [1]. Oligodendrogenesis consists in neuronal precursor cell (NPC) differentiation into the OPC lineage [11] that involves the upregulation of Sonic Hedgehog (Shh) and mitogenic factors, including epidermal growth factor (EGF) [3,10,12,13,14]. Canonical Shh signaling leads to the activation of the G-protein-coupled seven-pass transmembrane receptor Smoothened (Smo) that binds to the transcription factors Gli1-3, thus promoting Shh-mediated gene transcription. For unclear reason, Shh upregulation does not result in Gli1 upregulation after remyelination stimuli, with the consequence that NPCs are fated to the OPC lineage [10,12,13,14,15,16,17]. The expression of myelin regulatory factor (Myrf) marks the entrance of OPCs into the second phase of differentiation. Upon Myrf expression, OPCs mature into premyelinating OLs (Pre-OLs) that are characterized by a transcriptional profile that only partially overlaps with that of OPCs. Pre-OLs begin to express myelin proteins, one of the most abundant being the cytosolic MBP [3,18,19,20]. The observation that Myrf is poorly expressed in chronic MS lesions suggests that OPC maturation might be defective in SPMS patients [21].

The final step of OL maturation is axon engagement. At the peak of MBP expression, the OL membrane enlarges and flattens in a process that requires phosphatidylinositol 4,5 bisphosphate (PIP2) binding to MBP. This triggers the release of cofilin and gelsolin, two actin-binding proteins that regulate cytoskeleton depolymerization [22,23,24]. The observation that OPCs readily recognize inert polystyrene (PS) microfibers of the appropriate diameter (2–4 μm, i.e., similar to axons) opened the way to study the physical and molecular cues required for axon engagement in vitro and the efficacy of promyelinating drugs in the process of axon engagement [25,26,27,28,29]. How OLs recognize the presence of fibers in the three-dimensional (3D) space and how OL membrane rolling on fibers is stimulated remains a field of active study [23,24,30].

To investigate the signals promoting OPC differentiation into myelinating OLs and how axon engagement is stimulated in the absence of neuronal feedback, here we used as a toolkit Clobetasol and Gefitinib, two drugs that have been shown to promote remyelination in vitro and/or in vivo [8,9,31]. Molecular studies have shown that Clobetasol acts essentially via the glucocorticoid receptor (GR) [9] and the Smo receptor [8,32] to promote MBP expression during OPC to OL differentiation, while Gefitinib is an epidermal growth factor receptor (EGFR) tyrosine kinase inhibitor (EGFR-TKI) potentially acting on multiple receptors of the EGFR/ErbB family [33].

Several studies have indicated that EGFR/ErbB antagonists promote OPC differentiation in a demyelination environment but, due to the complex regulation of the EGFR/ErbB family of receptors at different steps of OL formation and under remyelination stimuli, they have not clarified how these compounds promote remyelination [8,34,35,36,37,38,39].

Using Gefitinib, EGFR gene silencing, alone or in combination with Clobetasol treatment and chambers containing PS microfibers (synthetic axons) to simulate OPC differentiation in vitro in a 3D environment, we show that Gefitinib, by inhibiting EGFR, promotes MBP expression and by inhibiting the PI3K/AKT pathway causes PIP2 re-localization to membranes, thereby initiating the events that lead to membrane expansion at the peak of MBP expression. Gefitinib in combination with Clobetasol, by co-regulating multiple receptors involved in remyelination, greatly increases single drug activity, providing the basis to develop remyelination therapies based on a combination of these drugs.

## 2. Materials and Methods

### 2.1. Cell Culture

The Oli-neuM line (Cellosaurus ExPASy CVCL_VL76) was obtained and cultured as previously described [8] and routinely tested for contamination. Briefly, cells were expanded (six to eight passages) in growth medium (GM) composed of DMEM (Corning Inc., New York, NY, USA) supplemented with 10% fetal bovine serum (FBS, Corning), 2 mM l-glutamine (Gibco™, Thermo Fisher Scientific, Waltham, MA, USA), 1% pen-strep (Gibco™), 1 mM sodium pyruvate (Gibco™), and 15 mM HEPES (Sigma-Aldrich, Merck KGaA, Darmstadt, Germany) at 37 °C in 5% CO_2_. Cells were grown until 80% confluence (approximately 3 × 10^6^ cells in a 100 mm dish), then detached using Trypsin/EDTA (0.5%, Sigma-Aldrich) and centrifuged for 5 min at 132× *g*. The pellet was resuspended in fresh GM and placed in new sterile dishes. Differentiation medium (DM) was GM supplemented with 1% N2-supplement (175020–01, Gibco™), 60 nM triiodothyronine (T3, Sigma-Aldrich), and 53.7 ng/mL progesterone (Sigma-Aldrich). Oli-neuM cells were maintained under antibiotic selection with 500 μg/mL geneticin (G418, Gibco™), which was added to both GM and DM.

### 2.2. Compound Treatments

Clobetasol (Prestw-781), Gefitinib (Prestw-1270) were purchased from Prestwick Chemical Library^®^ (http://www.prestwickchemical.com/prestwick-chemical-library.html). Drug stocks (100 mM) were pre-diluted to a concentration of 10 mM or 5 mM in dimethyl sulfoxide (DMSO) for Clobetasol and Gefitinib, and added at a final concentration of 5 μM or 1 μM in DM, respectively, so that final DMSO concentration did not exceed 0.5%. Optimal final concentrations were previously established according to MBP levels in titration experiments [8]. 5 µM Forskolin (F6886, Sigma-Aldrich), 1µM Wortmannin [40,41] and 5 µg/mL insulin [42] were used dissolved in DMSO (0.5%). Unless otherwise stated, drug treatments were administrated in differentiation media (DM) after 24 h from Oli-neuM seeding. We refer to “vehicle treatment” as the combination of DM plus 0.5% DMSO max (DM + DMSO). Culturing and time of drug treatments (48 h) were previously established to be optimal for MBP expression in Oli-neuM [8]. Unless otherwise described, Oli-neuM cells were treated with 1 µM Gefitinib, 5 µM Clobetasol or drug vehicle (0.5% max DMSO) for 48 h in parallel experiments and cultured in 6–12 for biochemical assays or in 96-well plates for immunofluorescence studies or Real Time PCR (qPCR) studies.

### 2.3. Gene Silencing and Transfection

Retinoid X Receptor gamma (RXRγ) and Epidermal Growth Factor Receptor (EGFR) gene silencing was performed using MISSION^®^ esiRNA Technology (Sigma-Aldrich; https://www.mpi-cbg.de/esiRNA/), which increases specificity due to the complexity of each MISSION^®^ esiRNA pool that shares the same on-target but differs in the sequence-dependent off-target signatures. Specifically, we used EMU004971 (RXRγ; CAT n. ENSMUSG00000015843, 200 ng/µL, Sigma-Aldrich) and EMU075311 (EGFR; Cat n. ENSMUSG00000020122, 200 ng/µL, Sigma-Aldrich). Two Scramble siRNAs (Scramble7 n:4234503 and Scramble2 n:4234507; Sigma-Aldrich), were used as silencing controls for all analyses. siRNA Scramble 2 and/or 7 were run along with the EGFR esiRNA treatment. Mean data for siScramble were obtained from at least n ≥ 3 biological replicates. Silencing was performed using a Reverse protocol: 3 µg of esiRNA per well were diluted in 200 µL of Opti-MEM^®^ (31985-070, 1X, Gibco^TM^) and after 5 min combined with Lipofectamine^®^ 2000 reagent (11668-09, Invitrogen™, Thermo Fisher Scientific) diluted in Opti-MEM^®^. The combined mixture was added after 20 min. Thereafter, 2.6 × 10^5^ Oli-neuM cells were seeded in 6-well plates in GM media and cells were grown and differentiated in DM after 24 h with or without treatment. Plasmid Transfection: Oli-neuM cells were seeded in growth chamber containing PS fibers for 24 h, treatment with indicated drugs and transfected with the PH-PLCδ-pEGFP plasmid [43] using Lipofectamine^®^ DNA Transfection Reagent Protocol (Invitrogen, Thermo Fisher Scientific). Plasmid amplification was performed as previously described [44] using GeneElute™ Plasmid Miniprep Kit (PLN70-1KT, Sigma-Aldrich) and the manufacture protocols. After extraction the plasmid were digested to confirm the presence of the specific insert described above.

### 2.4. Crude Extract Preparation and Immunoblot Analysis

Typically, 2.75 × 10^5^ Oli-neuM cells were seeded in 6-well plates in GM media and cells were grown to 70% confluence. Treatments, unless otherwise specified, were performed as indicated in the text, as previously described. For immunoblot analyses, the following antibodies were used: Cell Signaling Technology (Danvers, MA, USA): anti-phospho-p44/42 MAPK (Erk1/2Thr202/Thr204: #9101, 1:5000), anti-p44/42 MAPK (Erk1/2: #1240, 1:3000), anti-Phospho AKT (Ser473: #4060, 1:2500); anti-AKT (pan: #4691, 1:2000). Sigma-Aldrich: anti-actin (2066, 1:2000). AbD Serotec (Bio-Rad Laboratories, Hercules, CA, USA): anti-MBP (MCA409S, 1:200). Proteintech^®^ (Proteintech Group, Rosemont, IL, USA): anti-RXRγ (11129-1-AP,1:600). GeneTex (GeneTex, Inc., Irvine, CA, USA): anti-EGFR (GTX132810, 1:1000). Cell extract (CE) preparation and immunoblot analyses were performed as previously described [8]; briefly, bands signal intensity was estimated using ImageJ software (version 1.8.0), and data were plotted using GraphPad Prism 7.0 (GrahPad Software, San Diego, CA, USA) as the fold change versus vehicle, arbitrarily set to 1.

### 2.5. Quantitative Immunofluorescence Analysis and Confocal Microscopy

For IF quantitative analyses, Oli-neuM cells were plated in 96-well plates (655090, Greiner Bio-One, Kremsmünster, Austria), pre-coated with fibronectin from human plasma (Sigma-Aldrich) as previously described [44]. After 24 or 48 h (to obtain about 60% confluence), GM was removed and DM was added containing the indicated drug treatments or drug vehicle (0.5% DMSO). Unless otherwise specified, cells were further incubated for 48 h prior to fixation and processing for IF as previously described [8,44]. Acquisition was performed at 20× magnification (HCX PL FLUOTAR 20× NA 0.4) using a Leica DMI6000 B epifluorescence inverted microscope (Leica Microsystems, Wetzlar, Germany) equipped with Leica Application Suite X and Matrix Screener software (version 3.0) for automated image acquisition. Plate Array: vehicle-treated samples were spotted appropriately in different positions and acquired along with single drug treatments. Vehicle data (DM + DMSO 0.5%) of each plate was used to normalize signal intensity in the different channels in order to compare plate replicates. A minimum number of 1000 cells per sample was considered for mean intensity data evaluation of each treatment. Micrographs were analyzed with Scan^R^ (version 2.1; Analysis software version 1.1.0.6, Olympus, Tokyo, Japan) for quantification and statistical analyses as previously described [8,45]. Hoechst 33,342 (Invitrogen, Thermo Fisher Scientific) staining was used for nucleic acid quantification and nuclei detection. Rat anti-MBP (MCA409S; Serotec, 1:100), Rabbit anti-actin (A2066 Sigma-Aldrich, 1:80) primary antibodies and the respective Alexa Fluor 488 or Alexa Fluor 546 conjugated secondary antibodies (Thermo Fisher Scientific) were used as indicated in the text. Immunofluorescence (IF) and data quantification were performed as previously described [8,45]. Protein quantification in IF images was performed using the INTENSITY module of Scan^R^ analysis software: typically, the mean intensity FITC or TRITC values (±SEM) were detected on the mask MAIN of three biological replicates. One experimental replicate was constituted by 25 image/well, taken blindly by the acquisition software based on a 5 × 5 acquisition mask. Each sample was spotted in triplicate in each plate. Biological replicates that contained data from at least 1000 cells per sample were considered for statistical analyses. Quantitative morphological analysis was performed by using either EDGE or INTENSITY module of Scan^R^ analysis software (Olympus). We then calculated the percentage of the cell population with a higher membrane area using the Max Feret Diameter parameter of Scan^R^ software. The maximum Feret’s diameter is a measure of an object size along its maximal axis. By plotting the Max Feret Diameter along one axis (y) and the Mean Intensity FITC on the other axis (X) of a scattered plot it is possible to visualize the cell distribution accordingly. Specifically, the following parameters were used for gating the cell population of interest: Max Feret Diameter > 70 (y)/Mean Intensity FITC (MBP) > 300 (x), using Scan^R^ analysis software. This gate identifies the cell population that has higher membrane extension as well as higher MBP levels (FITC or TRITC channel according to the secondary antibody used). Three wells per sample of three biological replicates were acquired for each experimental condition and tested for statistical significance using Student’s *t* test vs. vehicle and one-way ANOVA for drug treatment comparisons.

To detect co-localization of MBP and PIP2, a LSM Fluoview 1000 confocal microscope (Olympus) equipped with an Olympus IX-81 inverted microscope was used. Microscopy inspection was performed at 48 h. Typically, Oli-neuM cells were seeded and treated on glass coverslips as indicated. After fixation, cells were alternatively incubated with FITC-conjugated anti-PIP2 (AC14-0106-12; Abcore, Ramona, CA, USA) or anti-MBP antibody, and stained with Hoechst 33342 to detect nuclei. Glass coverslips were mounted on a microscope slide using Fluoromount™ Aqueous Mounting Medium (Sigma-Aldrich). Micrographs were captured with a 20× UPLSAPO (NA 0.75, WD 0.65 mm) objective. For co-localization analysis, data were processed for 2D and 3D reconstruction using the isosurface tool of Imaris software (version 6.2.1, Bitplane, Zurich, Switzerland). Statistical coefficients (Pearson’s Correlation Coefficient; Mander’s Overlap Coefficient; Mander’s Co-localization Coefficient) were calculated for each region of interest (ROI) and then expressed as mean ± Standard Deviation (SD). PLCδ1PH-GFP was acquired using 488 nm laser line.

### 2.6. Evaluation of Cell Engagement on PS Microfibers

Cell culture chambers containing electrospun fibers were prepared as follows. Fibers were electrospun on 22 × 22 mm glass coverslips placed on a lab-built cylindric rotating collector (diam. 200 mm, linear velocity 12 m/s), starting from a 30% polystyrene (PS, MW 280 kDa, Sigma-Aldrich) solution in 1:1 tetrahydrofuran/dichloromethane (Sigma-Aldrich), using a flow rate of 0.5 mL/h from a 23G needle placed at a distance of 22 cm from the collector, and with a voltage of +15kV and −1 kV (CZE-2000, Spellman High Voltage, Hauppauge, NY, USA) applied to the needle and to the collector, respectively. Following electrospinning, PS fibers were immobilized using rectangular PDMS gaskets obtained as replicas from PMMA micromachined molds, and irreversibly bonded to glass coverslip surface following oxygen plasma activation (FEMTO plasma cleaner, Diener, Ebhausen, Germany). Chambers were UV-sterilized before use and pre-treated with 10 µg/mL fibronectin (F0895, Sigma-Aldrich). 90,000 Oli-neuM cells were seeded in growth medium and, after 24 h, medium was exchanged with either differentiation medium (DM) supplemented with 0.5% DMSO (vehicle) or with the indicated treatment(s). Cells were grown for 72 h at 37 °C in 5% CO_2_. After fixation, chambers were processed for immunofluorescence using the antibody indicated in the text. Acquisition was performed using a Leica DMI6000 automated microscope (Leica Microsystems) equipped with LAS-X Matrix Screener acquisition software (v. 3.0). Three positions per sample and 25 images (5 × 5 mosaic) for each position were considered for statistical analyses (n = 3). Engagement analyses were performed as described by Bechler et al., [25] with the following minor modification: nuclei located on fibers within a range of 86 µm around the fibers were considered engaged. The range of 86 µm was empirically chosen as it allows to distinguish cells occasionally near to fibers from those that effectively engage fibers, characterized also by MBP expression and elongated nuclei as described in the text. Images were visualized and analyzed with Scan^R^ (Olympus) or ImageJ [8,44] software tools as indicated above.

### 2.7. Total RNA Extraction and qPCR

Following drug administration, total RNA was extracted using TRI Reagent^®^ (T9424, Sigma-Aldrich) according to the manufacturer’s instructions. Typically, 2 µg of RNA sample were retro-transcribed using the High-capacity cDNA Reverse Transcription kit (4368814, Thermo Scientific) according to the manufacturer’s instructions. qPCR was performed using either SYBR Green- or TaqMan-based technology and the StepOne™ Real-Time PCR System (Applied Biosystems^®^, Thermo Fisher Scientific). Primer pairs used with StoS Quantitative Master Mix 2X SYBR Green-ROX (GeneSpin Srl, Milan, Italy) are reported in Table S2. Predesigned primer sets were used with TaqMan™ Gene expression MasterMix (4369016, Thermo Fisher Scientific): RXRγ (Mm00436411_m1), Glyceraldehyde 3-phosphate dehydrogenase (GAPDH) (Mm99999915_g1). GAPDH was used as endogenous control. Typically, 50 ng of cDNA per sample were used per reaction. qPCR was performed in triplicate in MicroAmp Fast Optical 48-Well Reaction Plate (Applied Biosystems^®^), n = 3. The ΔΔCT method of relative quantification was used to determine the fold change in expression. This was done by normalizing the resulting threshold cycle (CT) values of the target mRNAs to the CT values of the endogenous control GAPDH in the same samples (ΔCT = CT_target_ − CT_GAPDH_), and by further normalizing to the control (ΔΔCT = ΔCT − ΔCT_vehicle_). Fold change in expression was then obtained (2^-ΔΔCT^) and represented in the plots using a log_2_ scale for ease of visualization of up/down-regulated genes.

### 2.8. Bioinformatics and Statistical Methods

Chemical-protein network analysis was performed using STITCH software (http://stitch.embl.de/) using ver. 3.0 software and high score cut-off settings. Drug list used as input is shown in Table S1 and base on Najm et al. [9] and Porcu et al. [8]. In studies performed in multiwell plates (immunofluorescence and qPCR), three replicates per sample were spotted in each plate and the mean values obtained from the three samples were considered as one biological replicate. The mean values ±SEM obtained from at least three biological replicates were considered for statistical analyses. Statistical analyses were performed using GraphPad Prism. Effects of each drug treatment versus its internal control (vehicle) in immunofluorescence experiments, WB and qPCR data were analyzed using paired two-tailed Student’s *t* test, while one-way analysis of variance (ANOVA) with Tukey’s or Holm-Sidak post hoc tests (as indicated in figure legends) were used to determine statistically significant differences among multiple single or combined treatments.

## 3. Results

### 3.1. Gefitinib Promotes MBP Expression and Oli-neuM Differentiation in Myelinating OLs

**Figure 1:**
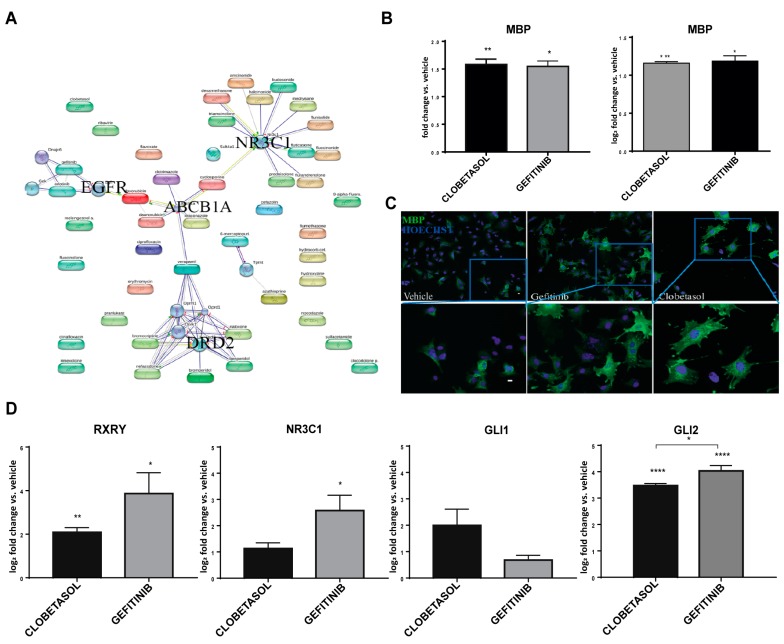
Gefitinib and Clobetasol stimulate MBP, RXRγ and Gli2 expression and Oli-neuM differentiation (**A**) STITCH Confidence view. Chemical-network analysis of drugs promoting MBP expression in OPC cell lines. Thicker lines indicate stronger associations; grey lines: protein-protein interactions. Green: chemical-protein interactions; red: interactions between chemicals. See Table S1 for chemical list and details. (**B**) MBP expression analysis of Oli-neuM treated for 48 h with 5 μM Clobetasol or 1 μM Gefitinib. Left panel: immunofluorescence quantitative analysis; Right panel: qPCR expression analyses. Data were normalized to vehicle, and are expressed as mean ± SEM (n = 3). Statistical significance: two-tailed paired Student’s *t* test was used for treatment versus vehicle. (**C**) Representative images of cells quantified in the left panel in B. The bottom images show enlargements of the boxed areas. Scale bar, 10 μm. (**D**) qPCR expression analysis of Oli-neuM treated with the indicated drugs. qPCR was performed using oligos indicated in Table S2. Oli-neuM cells were treated as in (**B**). Data were normalized to an internal control (GAPDH) and plotted as log_2_ fold change vs. vehicle, ±SEM using GraphPad Prism (n = 3). Log_2_-fold changes were used to show graphically the positive and negative changes in expression in a symmetrical manner. The level of expression in the control (which equals 1) is represented as 0. Statistical significance: two-tailed Student’s *t* test was used for treatment versus vehicle, ANOVA one-way with Tukey’s multiple comparison test was used to determine significance among different treatments. * *p* < 0.05, ** *p* < 0.01, *** *p* < 0.001, **** *p* < 0.0001.

Four main pharmacological classes have been identified in phenotypical screens for promyelinating agents based on MBP upregulation: glucocorticoids, EGFR/ErbB inhibitors, Benztropine/Bromocriptine and antifungal drugs such as Miconazole and Clotrimazole. We established a chemical–protein network using STITCH ver. 3.0 software (www.stitch.embl.de), using a high score cut-off setting. This analysis indicated the membrane-associated ATP-binding cassette (ABC) transporter ABCB1A (MDR) at the hub of all selected drug classes (Figure 1A; Table S1). As expected, the glucocorticoid receptor GR/NR3C1 was found at the node of the glucocorticoid group of compounds [8] and the EGFR/ErbB receptors at the node of Gefitinib and Erlotinib drug action. Surprisingly, Benztropine and Bromocriptine form a group with DRD2/3 dopaminergic receptors as common targets (Figure 1A, Table S1). Previous work associated the effect of Benztropine on OPC differentiation with its activity on the M1/M2 muscarinic receptor [5]. More recently, the effects of Miconazole, Clotrimazole and Benztropine action on MBP expression has been attributed to their common ability to inhibit two key enzymes of the cholesterol biosynthetic pathway leading to 8,9-unsaturated sterol accumulation [46]. How these different observations can be reconciled remains to be investigated [47].

Here, we focused on validating the predicted role of the EGFR/ErbB antagonists in OPC maturation up to the stage of axon engagement. Gefitinib is a well-characterized anticancer agent that targets multiple membrane receptors of the EGFR/ErbB family, with a higher affinity for the ATP binding pocket of the EGFR receptor [33,48]. The glucocorticoids Clobetasol, Fluorandrenolide, Halcinonide, Amcinonide and Medrisone are characterized by agonistic activity on the Hedgehog pathway via Smo [32], in addition to their activity on the glucocorticoid receptor [49]. The requirement of Smo activation for MBP expression in Clobetasol-treated Oli-neuM cells was previously confirmed experimentally [8].

To compare the effects of Gefitinib and Clobetasol on MBP expression in Oli-neuM, we performed quantitative immunofluorescence microscopy (IF) and qPCR analyses (Figure 1B). Treatment of Oli-neuM cells with either Gefitinib or Clobetasol confirmed that they are similar in their ability to increase MBP levels compared to the vehicle control (Figure 1B). Given that MBP mRNA is co-transported with ribonucleic particles to endosomes prior to being translated locally [50,51], we tested whether MBP upregulation was due to increased gene transcription and/or increased translation. Using qPCR, we observed that Clobetasol or Gefitinib treatment also enhances MBP gene transcription in addition to increasing MBP protein expression (Figure 1B, right panel).

To test if this increase in MBP was paralleled by a progression in the differentiation of the cells to an OL-like stage, we monitored the morphology of Oli-neuM cells after Gefitinib or Clobetasol treatment using immunofluorescence microscopy. Differentiated OLs have enlarged and flattened membranes filled with MBP (Figure 1C) [24,50,51]. Oli-neuM cells pass from a triangular or round shape, typical of pre-OLs, to an enlarged and flattened morphology, typical of myelinating OLs. In cells with enlarged membranes, MBP filled the cytosol as well as the digit processes where the brightest spots of MBP were visualized, indicating that the protein accumulates at these sites (Figure 1C and see below for quantitative morphological analyses).

We concluded that Gefitinib and Clobetasol similarly promote MBP expression and Oli-neuM differentiation from a pre-myelinating stage to a differentiated morphology typical of myelinating OLs.

### 3.2. Gefitinib and Clobetasol Treatment Upregulate RXRγ and Gli2 Expression

The essential players required for pre-OL differentiation into mature OLs are poorly understood [3]. Oli-neuM express Myrf [8,19,52]; thus, we concentrated our attention on nuclear factors acting downstream of Myrf expression. Of these, the 9-cis retinoic acid-responsive element RXRγ is known to be upregulated at the transition from NPCs to the OPC lineage [53], as well as at lesions in the lysolecithin-induced murine demyelination model [54]. Furthermore, we have previously shown that the RXRγ inhibitor UV3003 downregulates MBP expression upon Clobetasol or Halcinonide treatment of Oli-neuM [8]. This indicates that the OPC transition to myelinating OLs is recapitulated in Oli-neuM under Clobetasol treatment via RXRγ expression. However, the signal that promotes RXRγ expression under remyelination stimuli or Clobetasol treatment remained to be determined [54,55].

To determine if RXRγ expression is similarly regulated under Gefitinib or Clobetasol treatment, we measured the expression of RXRγ along with that of Smo effectors Gli1 and Gli2 [56] or the GR NR3C1 [8] using qPCR and the oligos indicated in Table S2.

We confirmed that Clobetasol stimulates RXRγ expression without significantly altering Gli1 or NR3C1 expression (Figure 1D, respective panels) as previously reported [8]. Gefitinib treatment significantly up-regulates both RXRγ and NR3C1, but not Gli1 (Figure 1D, respective panels). Of note, either Gefitinib or Clobetasol treatment potently enhance Gli2 gene transcription (Figure 1D, respective panels).

Thus, Gefitinib and Clobetasol share the ability to promote expression not only of MBP, but also of Gli2 and RXRγ, while they poorly affect the expression of the canonical Smo target, Gli1. Since Gefitinib treatment has not been previously associated with RXRγ or Gli2 expression, we hypothesized that this is a specific function that the EGFR/ErbB inhibitor exerts in developing OPCs that might be required to promote their transition to myelinating OLs.

**Figure 2:**
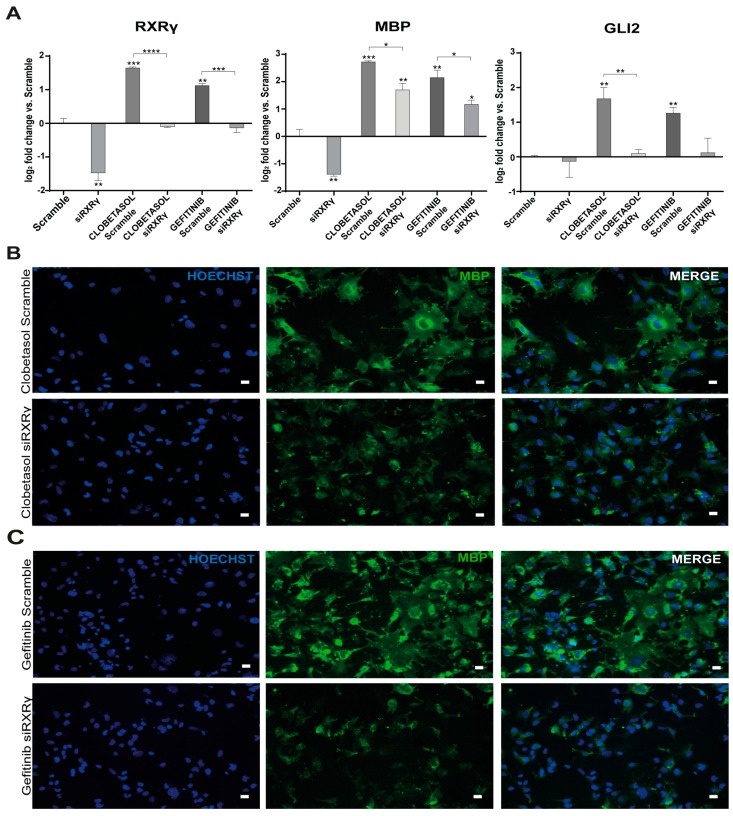
RXRγ gene silencing reduces MBP expression under Clobetasol or Gefitinib treatment. (**A**) qPCR analyses of RXRγ, MBP, and Gli2 gene expression in Scramble-silenced (Scramble) or RXRγ-silenced (siRXRγ) Oli-neuM cells. Cells were seeded and treated as indicated in Materials and Methods. After 48 h mRNA was extracted and processed for qPCR using oligos indicated in Table S2. Data were normalized to an internal control (GAPDH) and plotted as log_2_ fold change vs. control (mean of siScramble 2 + 7) ± SEM (n ≥ 4) using GraphPad Prism. Log_2_ fold changes were used to show graphically the positive and negative changes in expression in a symmetrical manner. The level of expression in the control (which equals 1) is represented as 0. Statistical significance: two-tailed paired Student’s *t* test was used for treatment versus control. One-way ANOVA with post-hoc Tukey was used for comparing multiple treatments. * *p* < 0.05, ** *p* < 0.01, *** *p* < 0.001, **** *p* < 0.0001. (**B**) Representative images of siScramble-silenced or siRXRγ Oli-neuM cells treated for 48 h with Clobetasol or (**C**) Gefitinib. Immunofluorescence analyses were performed with anti-MBP and nuclei were stained with HOECHST (Blue) as indicated in Materials and Methods. Scale bar: 10 μm.

To determine the importance of RXRγ for Gefitinib- or Clobetasol-mediated MBP and/or Gli2 expression, we performed epistatic studies by silencing RXRγ expression using esiRNA (see Materials and Methods and Table S2) and measuring Gli2 and MBP expression in RXRγ-silenced cells after drug treatment. All data were evaluated against treated siScramble-transfected cells as control (Figure 2). RXRγ silencing completely abrogated the stimulatory effects of Clobetasol or Gefitinib on RXRγ gene transcription (Figure 2A, RXRγ panel). Furthermore, we observed that Clobetasol or Gefitinib treatment of RXRγ-silenced cells significantly reduced MBP expression over the siScramble control as determined by qPCR (Figure 2A; MBP panel), immunoblot (Figure S1, respective panels) and immunofluorescence analyses (Figure 2B, panels Clobetasol and, 2C, panels Gefitinib, respectively). Despite RXRγ being heavily down-regulated upon silencing (average silencing was about 80%, Figure S1), the reduction in Gli2 expression levels was statistically significant only for Clobetasol-treated but not for Gefitinib-treated cells, compared to controls (Figure 2A panel Gli2).

We concluded that MBP upregulation requires RXRγ expression after Clobetasol or Gefitinib treatment, and thus RXRγ expression is an essential component of the signaling that leads to MBP expression in Oli-neuM under these treatments. The requirement of RXRγ for Clobetasol-mediated Gli2 expression supports the view that Clobetasol treatment acts through multiple receptors in Oli-neuM differentiation, one of which is Smo, as we previously suggested [8].

### 3.3. EGFR/ErbB Inhibition Promotes MBP Expression and Gefitinib or siEGFR Silencing Combined with Clobetasol Treatment Potently Stimulates MBP Expression and Oli-neuM Differentiation

**Figure 3:**
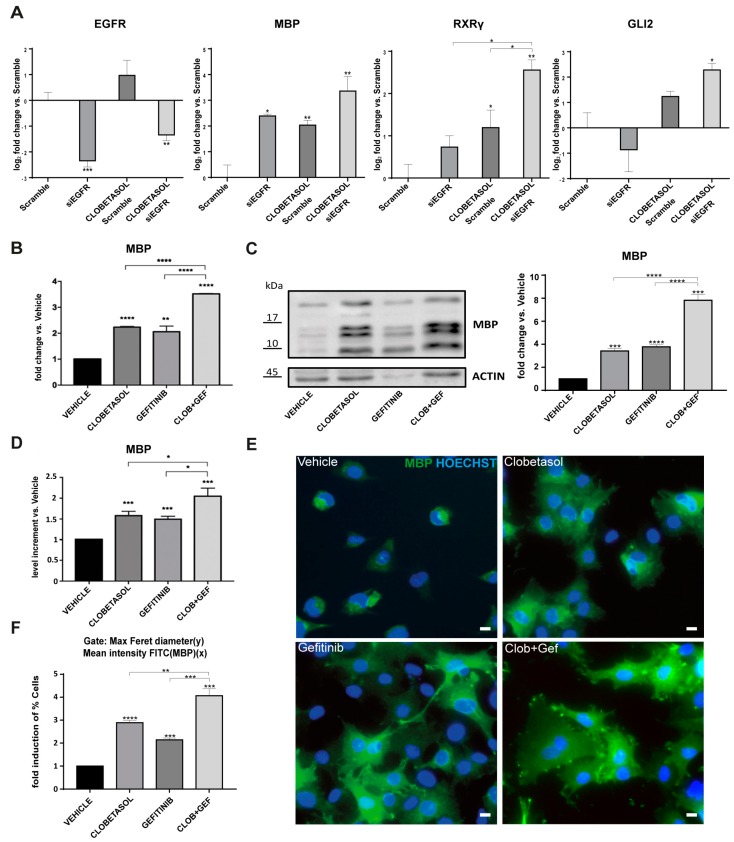
Epidermal growth factor receptor (EGFR) gene silencing upregulates MBP expression and increases Clobetasol-mediated Oli-neuM differentiation. (**A**) qPCR analysis of siEGFR Oli-neuM cells versus control (siScramble) in untreated or Clobetasol-treated Oli-neuM. The graphs show gene expression levels of EGFR, MBP, RXRγ and Gli2 (respective panels) after 48 h treatment. Data were plotted as log_2_ fold change versus control (siScramble) ± SEM (n ≥ 4) Log_2_-fold changes were used to show graphically the positive and negative changes in expression in a symmetrical manner. The level of expression in the control (which equals 1) is represented as 0. (**B**–**F**) Comparison of 1 μM Gefitinib or 5 μM Clobetasol single or combinatorial drug effects: (**B**) qPCR analyses of MBP gene expression under the indicated treatments versus vehicle (DM + DMSO). Data were normalized to an internal control (GAPDH) and plotted as fold change induction above vehicle arbitrarily set as 1. Data are mean ± SEM (n = 3). (**C**) Immunoblot analyses of MBP expression with the indicated treatment. Left panel: Representative immunoblot analysis using anti-MBP or anti-Actin antibody as indicated in Material and Methods; right panel: quantification of bands detected in immunoblots (n = 3 ± SEM) using ImageJ tools. Data are plotted as fold induction versus vehicle arbitrarily set at 1; (**D**) Immunofluorescence quantitative analyses of images of Oli-neuM cells treated as indicated. Mean intensity FITC (MBP) was plotted as mean ± SEM (n = 5). Data were normalized to vehicle, arbitrarily set to 1, and plotted as fold induction versus vehicle. (**E**) Representative images of Oli-neuM vehicle-treated cells stained with α-MBP primary and Alexa 488 secondary antibodies (FITC); HOECHST (Blue) = nuclei. Scale bar, 10 μm. (**F**) Quantitative morphological analysis was performed using Scan^R^ software (Olympus) using maxFeretDiameter and Mean Intensity FITC parameters. Data were plotted in a graph by Scan^R^ software and the mean % population within the gate MaxFeretDiameter > 70 (y)/MeanFITC (MBP) (x) comparatively evaluated to identify the treatment promoting higher MBP expression and larger membrane expansion. Data are shown as fold induction versus vehicle arbitrarily set at 1. Statistical significance was calculated using GraphPad: two-tailed Student’s *t* test versus control (siScramble Figure 3A) or versus Vehicle (Figure 3B–F). One-way ANOVA with Tukey’s correction was used for multiple treatment comparison. * *p* < 0.05, ** *p* < 0.01, *** *p* < 0.001, **** *p* < 0.0001.

Since Gefitinib has a higher affinity for the ATP pocket of EGFR compared to other targets in the EGFR/ErbB family [33,48], we wondered if inhibition of this specific receptor regulates MBP and/or RXRγ expression. For this we used EGFR esiRNA (siEGFR; see Materials and Methods) and found that MBP gene transcription is significantly upregulated by EGFR silencing compared to siScramble-transfected cells (Figure 3A). While a slight increase in RXRγ gene expression and a slight reduction in Gli2 gene expression compared to controls were observed in EGFR silenced cells, these effects were not statistically significant (Figure 3A respective panels). These data are consistent with the idea that EGFR inhibition per se favors MBP expression, but that co-regulation of multiple receptors is at the basis of RXRγ and Gli2 gene expression under Gefitinib treatment.

These observations suggested that MBP expression might be further increased relative to other treatments by appropriately co-regulating EGFR (inhibition) and the Clobetasol receptor targets. Cooperative signaling responding to Smo and EGFR receptor co-activation was previously observed in medulloblastoma cells treated with Gefitinib [57] and Clobetasol action on MBP has been attributed to Smo [8] and/or GR [9] activation. Therefore, we wondered if the Clobetasol promyelinating activity could be further enhanced by EGFR gene silencing. To validate this idea, we treated EGFR silenced Oli-neuM cells with Clobetasol and measured MBP expression after 48 h by qPCR. Confirming a potential crosstalk between Smo and EGFR signaling pathways, silencing of EGFR under Clobetasol treatment potently stimulated MBP compared to controls (Figure 3A).

These data prompted us to evaluate the efficacy of the combinatorial treatment of Clobetasol and Gefitinib on MBP expression and on Oli-neuM differentiation.

Oli-neuM cells were treated with Clobetasol and Gefitinib (CLOB + GEF), with each drug alone, or with vehicle controls for 48 h and the effects on MBP expression were determined using qPCR (Figure 3B), by immunoblot analyses (Figure 3C), and by quantitative immunofluorescence microscopy (Figure 3D). Clearly, MBP expression is significantly enhanced at both the mRNA and protein level in co-treated cells compared with single treatments or vehicle controls. Accompanying the MBP increase, as expected, cells maximize their membrane size (Figure 3E), as determined by quantitative morphological analyses of the Max Feret Diameter of cells expressing high amounts of MBP (Figure 3F).

Collectively, these data show that EGFR inhibition per se stimulates MBP and partially RXRγ expression in pre-myelinating Oli-neuM. Importantly, when combined with Clobetasol treatment, potently stimulates not only MBP expression but also OPC differentiation by unlocking the events that lead to membrane expansion. Independently confirming this view, Gefitinib, which inhibits EGFR but also other members of this receptor family, not only promotes MBP expression and membrane expansion but also up-regulates RXRγ and Gli2 gene transcription.

### 3.4. Clobetasol and Gefitinib Co-Treatment Enhances Oli-neuM Differentiation by Co-Regulating PIP2 Availability with MBP Expression

It is well-described that Gefitinib inhibits the phosphatidylinositol-3-kinase (PI3K) signaling pathway and impairs proliferation in tumor cells [51]. PI3K uses PIP2 as a substrate to produce phosphatidylinositol (3,4,5)-trisphosphate (PIP3) [58], which is required to activate the AKT-dependent axis, which, when activated, promotes cell proliferation. However, how Gefitinib might promote membrane enlargement in OPCs by downregulating the PI3K/PIP3/AKT axis remains to be established. We reasoned that if Gefitinib downregulates the PI3K/PIP3/AKT signaling axis, it could (indirectly) favor an increase in PIP2 levels and/or its re-localization at OL membranes. Such an event could promote membrane enlargement by promoting PIP2/MBP complex formation [23,24]. Alternatively, Gefitinib treatment could promote differentiation by downregulating Ras/MAPK/ERK pathways.

**Figure 4:**
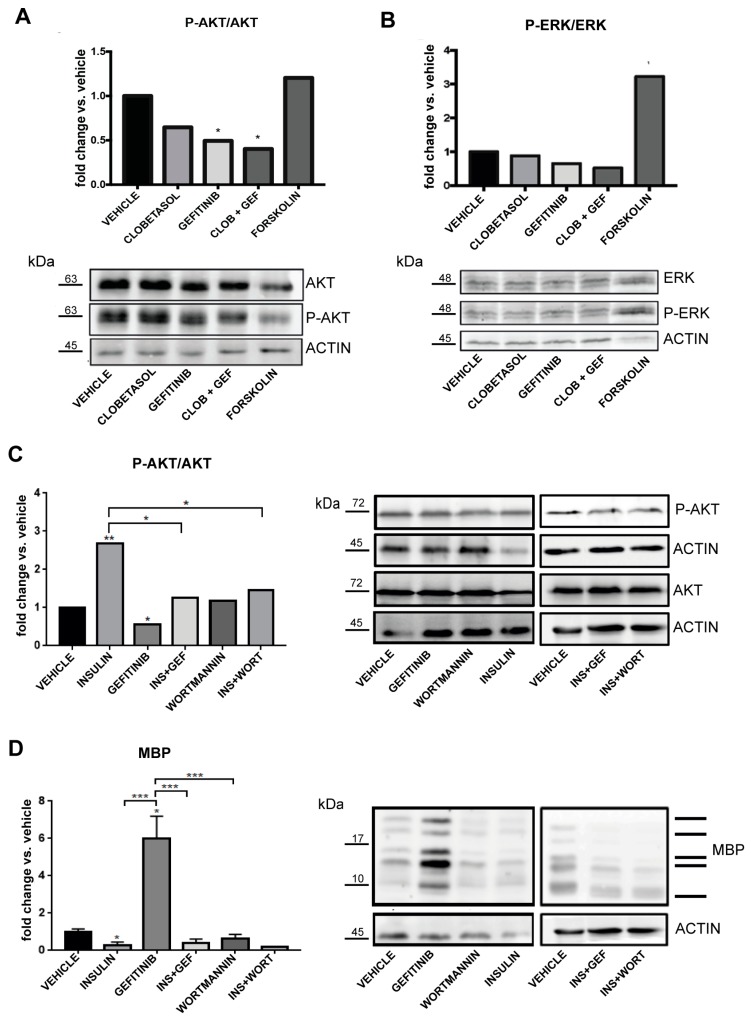
Analysis of the signaling pathways activated by Gefitinib treatment in Oli-neuM cells. (**A**,**B**) Immunoblot analysis Oli-neuM cell extracts after treatment with 1 μM Gefitinib, 5 μM Clobetasol, 5 μM Forskolin (as control treatment), or vehicle (DM + 0.5% DMSO). Quantitative analysis of protein levels: (**A**) top panel: anti-AKT (AKT) or phospho-AKT (P-AKT) antibody. Anti-actin antibody was used to normalize sample loading. (n = 3). Signal intensity was estimated on bands using ImageJ software tools, and data were plotted using GraphPad Prism as the fold change of P-AKT/AKT. A 2-fold change threshold versus vehicle was applied (indicated with *). Lower panel: representative image of immunoblots used for quantitative analysis. (**B**) Top panel: anti-ERK (ERK) or anti-phospho-ERK (P-ERK) antibody. Data and statistical analyses (n = 3) were performed as in (**A**). Lower panel: representative image of immunoblots used for quantitative analysis. (**C**,**D**) Immunoblot analysis of Oli-neuM cell extracts after treatment with insulin (5 µg/mL), Gefitinib (1 μM), Wortmannin (1 µM), insulin+Gefitinib (INS + GEF), Wortmannin+Gefitinib (WORT+GEF), or vehicle (DM + 0.5% DMSO). (**C**) Left panel: quantitative analyses of the bands obtained after immunoblotting using an anti-AKT (AKT) or phospho-AKT (P-AKT) antibody (n = 3). An anti-actin antibody was used to normalize sample loading (n = 3). Signal intensity was estimated using ImageJ software, and data were plotted using GraphPad Prism as the fold change versus vehicle, arbitrarily set to 1. Statistical significance: two-tailed Student’s *t* test was used for treatment versus vehicle, ANOVA one-way with Tukey’s multiple comparison test was used to determine significance among different treatments. * *p* < 0.05, ** *p* < 0.01, *** *p* < 0.001, **** *p* < 0.0001. Right panel: representative immunoblot image of treatments. (**D**) Left panel: quantitative analysis of bands visualized by immunoblotting using an anti-MBP antibody. Data and statistical analyses were performed as in (**C**). Right panel: representative immunoblot images.

We first asked if the PI3K/PIP3/AKT or Ras/MAPK/ERK pathways are affected under our experimental conditions. Oli-neuM cells were treated with Gefitinib alone or in combination with Clobetasol and the ratio of phospho-ERK1/2 (pERK) versus ERK or phospho-AKT (pAKT) versus AKT levels was determined by Western blot analysis. Forskolin was used as a positive control for MAPK/ERK activation [59], insulin as a positive control for PI3K/PIP3/AKT activation [42], and Wortmannin as a negative control for PI3K activation [40,41]. The results indicated that Gefitinib significantly inhibits the PI3K/PIP3/AKT axis (Figure 4A) over the MAPK/ERK axis (Figure 4B) under the conditions used in this study. Clearly, although Wortmannin antagonizes PI3K (Figure 4C) and, as expected, impairs insulin-mediated AKT activation when given in co-treatment (Figure 4C, INS + WOR), it does not promote MBP expression, unlike Gefitinib (Figure 4D, respective sample). Gefitinib, as expected, inhibits insulin-mediated AKT phosphorylation and conversely insulin completely abrogates MBP expression under Gefitinib treatment by bypassing Gefitinib-mediated PI3K inactivation. Together these data show that MBP expression under Gefitinib treatment does not depend only on PI3K/PIP3/AKT signaling inhibition but that PI3K activation can turn off Gefitinib-mediated MBP expression, supporting the multi-receptor nature of Gefitinib-dependent MBP expression.

**Figure 5:**
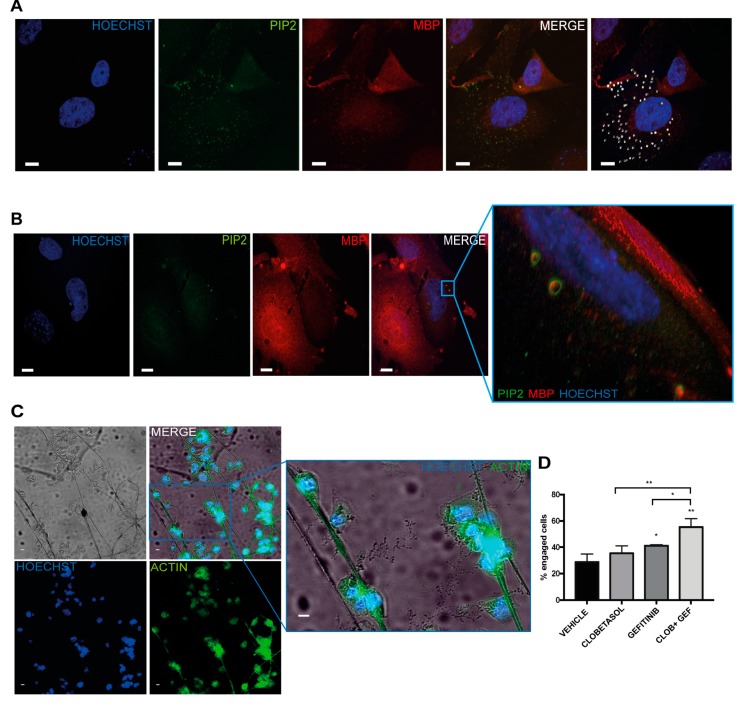
Clobetasol and Gefitinib co-treatment triggers MBP and PIP2 co-localization and enhances Oli-neuM engagement of polystyrene microfibers. (**A**,**B**) Confocal microscopy images of Oli-neuM cells treated with 5 μM Clobetasol and 1μM Gefitinib, stained with α-MBP (TRITC) and α-PIP2 (FITC). Nuclei (Hoechst). (**A**) Immunofluorescence images of cells showing PIP2 and MBP co-localization. Co-localization measurement details are shown in Figure S3. In the right-most panel, the co-localizing spots are highlighted by white dots. (**B**) Co-localization of MBP-PIP2. Colocalization analyses are in Figure S2. Confocal images of cells in which three-dimensional (3D) reconstruction has been performed. The blue boxed area in the MERGE panel indicates the region containing the co-localizing spots that is enlarged in the right panel. Blue boxed right panel: 3D volume reconstruction was performed using Imaris 6.21 suite on cropped images (Z-stack = 0.3 μm step size). Scale bar = 10 μm. (**C**) Epifluorescence IF microscopy images of Oli-neuM cells grown on PS microfiber chambers. Representative IF images of Oli-neuM cells treated for 72 h with 5 μM Clobetasol and 1 μM Gefitinib (CLOB GEF) cultured on supports containing electrospun PS microfibers (2–4 µm). Blue box shows an enlarged view of the region containing engaged cells. Cells were fixed and processed for IF microscopy as indicated in Materials and Methods. Fibers were visualized using phase contrast. Anti-actin (FITC), nuclei (HOECHST). Image acquisition was performed using a 20× objective, as indicated in Materials and Methods. Scale bar: 10 μm. (**D**) Engagement quantification after treatments. Image quantification was performed by analyzing 75 images/sample for each treatment (n = 3). Specifically, the percentage of engaged cells was estimated by counting nuclei located on fibers or within a range of 86 μm from the fiber. Data were plotted in the graph (n = 3, mean ± SEM) using GraphPad Prism as indicated in the text. Two-tailed paired Student’s *t* test was used for statistical significance versus vehicle (NT), one-way ANOVA with Tukey’s multiple comparison test was used to analyze statistical significance among different treatments. * *p* < 0.05, ** *p* < 0.01.

We reasoned that the Gefitinib treatment, by inhibiting the EGFR/PI3K/PIP3 pathway, might favor PIP2 re-localization at membranes and, thereby, PIP2 and MBP complex formation. As previously mentioned, the formation of the PIP2 and MBP complexes at the peak of MBP expression, by causing the release of the F-actin depolymerization proteins cofilin and gelsolin, promotes OL membrane enlargement [22,24,60]. To determine the existence of PIP2/MBP complexes in Clobetasol and Gefitinib co-treated Oli-neuM cells, we employed immunofluorescence confocal microscopy (see Materials and Methods) and anti-PIP2 and anti-MBP primary and the appropriate secondary antibodies. The PIP2/MBP complexes were visualized on acquired images by determining the Mean Pearson’s correlation coefficient (PCC) and the mean Mander’s overlap coefficient (MOC) of PIP2-(FITC) and MBP-(TRITC) signals in samples of treated compared to controls (Figure 5A; Figure S2). The resulting scatterplot of pixel intensity of co-localizing spots showed a clear correlation between PIP2 (green) and MBP (red) channels (n = 10). To visualize the structures to which these signals belong, a 3D surface reconstruction was performed using Imaris analysis tools (Figure 5B, blue box). This analysis showed vesicle-like carriers of about 600 nm containing PIP2 and MBP in the cytosol of Gefitinib and Clobetasol-treated Oli-neuM cells.

**Figure 6:**
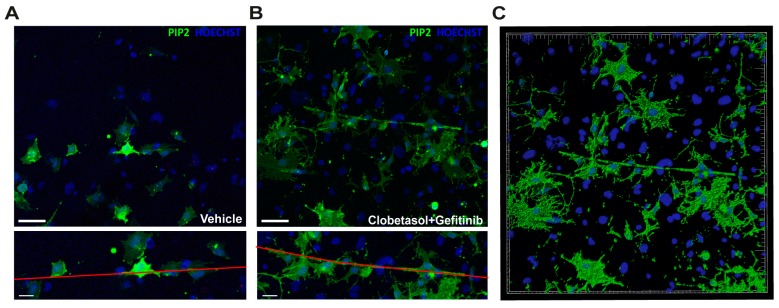
Clobetasol and Gefitinib promote lateral lengthening onto PS microfibers compared to vehicle-treated cells. Confocal microscopy images of PH-PLCδ-pEGFP-transfected Oli-neuM cells grown for 48 h in PS microfiber-containing growth chambers treated with (**A**) Vehicle (DM+ DMSO) or (**B**) 5 µM Clobetasol and 1 µM Gefitinib. Top (**A**,**B**) panels: confocal images, PIP2 (Green), Nuclei (Blue) Scale bar = 70µm. Lower (**A**,**B**) panels: detail of top images with enlargement of the cells engaging the PS microfiber. PS Microfibers are highlighted in red. Scale bar = 30 µm. (**C**) Volume reconstruction of image in panel (**B**), performed with Imaris software as described in Materials and Methods. Green = PIP2-containing membranes, blue = nuclei.

To establish if Oli-neuM cells treated with Clobetasol and Gefitinib had reached the final stage of differentiation that allows myelination of axons, we used cell culture chambers containing PS electrospun microfibers of 2–4 µm (PS chambers; Figure S3) suitable for microscopy inspection. This diameter of PS microfiber, by mimicking axons, allows primary OPCs to wrap along the fibers [25,26,27,28,46]. In our setup, Oli-neuM cells were seeded in the PS chamber 24 h prior to treatment (see Materials and Methods). Specifically, to establish the level of engagement of cells, we counted the proportion of cells with cytosol filled with MBP wrapped on the fibers (visualized in phase contrast) that also showed elongated nuclei (Hoechst; Figure 5C). Quantitative analysis of images showed that combined Clobetasol and Gefitinib treatment increases the number of Oli-neuM cells able to engage synthetic axons, compared to single or control treatments (Figure 5D). To determine the localization of PIP2 in Gefitinib and Clobetasol-treated cells during fiber engagement, we transfected Oli-neuM cells with plasmids carrying the pleckstrin homology (PH) domain of PLCδ1 fused to GFP (ΔPLCδ-pEGFP). The PLCδ1-PH domain is specifically recognized by PIP2 and thereby allows the visualization of PIP2 in fixed and living cells [43]. Clearly, PIP2-enriched membranes (Figure 6, green signals) wrap fibers over a long stretch in the Clobetasol and Gefitinib treated cells compared to vehicle control (Figure 6A–C), consistent with the role of Gefitinib and Clobetasol as remyelinating drugs.

## 4. Discussion

The current study shows that EGFR inhibition per se favors MBP expression in pre-myelinating OLs and Gefitinib, by inhibiting EGFR as well as other EGFR/ErbB family members, is a potent promyelinating agent. Clobetasol and Gefitinib co-treatment, by co-regulating at least three of the receptors involved in OPC differentiation, namely Smo, GR (activation) and EGFR/ErbB (inhibition), mimic the natural signals promoting OPC differentiation until axon engagement. We show that Clobetasol and Gefitinib co-treatment maximizes MBP expression and PIP2 co-localization at endosome-like structures with consequent membrane expansion allowing synthetic axon engagement.

This work initiated with the in silico analysis of the chemical-protein network of drug targets that emerged from the study of Porcu et al. [8]. This analysis indicated the existence of a cross-talking network of receptors involved in Oli-neuM differentiation. We hypothesized that if appropriately stimulated/inhibited by the promyelinating drug, each receptor would promote signaling pathways leading to OPC differentiation. Based on these hypotheses, we began by studying the effects of EGFR/ErbB receptor(s) regulation by Gefitinib or EGFR gene silencing.

Using Gefitinib, we have clarified that EGFR/ErbB receptor(s) inhibition has two main effects: on the one hand, it contributes to upregulating MBP, RXRγ and Gli2 gene expression, while on the other hand, by downregulating the PI3K/PIP3/AKT signaling cascade at the peak of MBP expression, it enhances PIP2 and MBP interaction and thereby membrane expansion. We observed that while Gefitinib upregulates RXRγ and Gli2 gene transcription it has little effect on the expression of NR3C1 and Gli1. By comparing EGFR gene silencing with Gefitinib treatment we found that EGFR silencing contributes to upregulating MBP gene expression, as does chemical inhibition, but less effectively enhances RXRγ gene transcription and poorly affect Gli2 gene expression. Thus, EGFR genetic inhibition does not completely overlap Gefitinib action on OPC differentiation. We concluded that Gefitinib acts through EGFR inhibition but also through other pathways in promoting OPC differentiation.

Using RXRγ gene silencing, we showed that RXRγ is necessary to maximize MBP expression under Gefitinib or Clobetasol treatment. However, RXRγ silencing does not abolish either Gefitinib- or Clobetasol-mediated MBP expression. Thus, both drugs act on MBP expression via multiple pathways, one of which requires RXRγ.

How Gefitinib or Clobetasol treatment leads to RXRγ upregulation remains to be determined in our or in other systems [54]. The fact that the combined treatment with Clobetasol and siRXRγ returns Gli2 expression to basal levels shows that RXRγ acts before Gli2 in Clobetasol-mediated signaling to MBP and confirms that Clobetasol acts not only by GR mediated phosphorylation on MBP expression [9], but also via a RXRγ/Gli2/MBP pathway as we previously suggested [8].

A correlation between RXRγ upregulation and Gefitinib treatment has been previously indicated by the cooperative antitumor activity of the RXRγ-selective agonist Bexarotene and Gefitinib in preclinical models of non-small cell lung cancer [61]. PI3K inactivation by Gefitinib treatment can promote Retinoic Acid Receptor (RAR) gamma 2 transcriptional activity in cancer cells [62]. Thus, Gefitinib treatment might indirectly contribute to RXRγ upregulation by stimulating RXRγ dimerization with other nuclear factors, among which RARγ [55]. Moreover, RARβ, the vitamin D receptor (VDR), and the peroxisome proliferator activated receptor (gamma PPARγ) have been co-immunoprecipitated with RXRγ in cell lysates from OPC or OL primary cells, indicating that they might also be the active heterodimers during the OPC to OL differentiation [63]. In addition, other nuclear receptors regulating lipid homeostasis in myelinating OLs, such as Liver X Receptor (LXR), orphan Nur77 and Nurr1, and Farnesoid X receptor (FXR), can form dimers with RXRγ [55]. Thus, RXRγ upregulation by Gefitinib or Clobetasol treatment might contribute to coordinating lipid metabolism with MBP expression under EGFR and Smo signaling in myelinating OLs.

Several members of EGFR/ErbB have been previously shown to be involved in PNS and/or CNS myelination [34,35,36,64] and the role of EGF in promoting oligodendrogenesis till OPC differentiation has been recently established [36], but the signal that promotes EGFR/ErbB inhibition during OPC differentiation remains unclear [34,35,64,65,66,67]. Unfortunately, ErbB2, ErbB3, or ErbB4 mouse knock out models have not allowed to clarify the role of each receptor in brain development: they are either embryonic lethal or show postnatal death with neurodevelopmental and cardiac defects [64]. This work adds EGFR to the list of EGFR/ErbB family members potentially regulating MBP expression in differentiating OPC, by placing EGFR functional inhibition as required after Myrf expression to enhance MBP expression and to promote its interaction with PIP2. Further study will clarify the role of EGFR, if any, in OL engagement.

Clearly, Gefitinib, which inhibits multiple members of the EGFR/ErbB family [33], might act on RXRγ and Gli2 gene expression via regulation of one of the other of the EGFR/ErbB family members. Among the EGFR/ErbB family members that might participate in OPC differentiation, the ErbB2 receptor could be a potential partner of EGFR. ErbB2 is an orphan receptor regulated by heterodimerization with other ErbB family receptors, and is known to interact with the negative regulator of myelination, LINGO 1 [63]. It will be interesting to study EGFR and ErbB2 co-regulation during OPC differentiation. Moreover, it cannot be excluded that the role of EGFR in OPC differentiation is metabolic. In fact, it has been shown that the EGFR/src signaling pathway regulates volume-sensitive organic osmolyte efflux pathways in astrocytes and EGFR or PI3K inhibition by AG1478 or Wortmannin, respectively, results in reduced efflux of Taurine [68]. Taurine recently emerged from a metabolomics screening study as a compound able to promote OPC differentiation alone or in combination with Benztropine [69].

Consistent with the hypothesis that PI3K downregulation by Gefitinib does not promote MBP expression per se, Wortmannin, which specifically inhibits PI3K, does not have any effect on MBP expression. Furthermore, insulin, which stimulates the PI3K/PIP3/AKT axis [70], completely abrogates Gefitinib-mediated MBP expression in Oli-neuM. By downregulating EGFR/PI3K/PIP3/AKT signaling Gefitinib promotes PIP2 re-localization leading to a further increase of MBP expression compared to EGFR inhibition alone.

Using 3D reconstruction studies, we were able to visualize endosomal-like structures containing both PIP2 and MBP in Gefitinib and Clobetasol co-treated Oli-neuM cells. We observed that PIP2 accumulates at the tips of processes in 2D culture and is enriched at the sites of fiber engagement when cells are grown in 3D supports. Given the role of PIP2 in the re-localization of receptors, including Smo, and cytoskeleton proteins to lipid rafts [71], it is tempting to speculate that the PIP2 enrichment at engaging membranes is the starting signal promoting wrapping during myelination.

In summary, our data support the view that Gefitinib, by inhibiting EGFR, promotes MBP expression and PIP2-mediated membrane enlargement, while other pathways regulate RXRγ gene expression under Clobetasol and Gefitinib treatment. Gefitinib, via PI3K/AKT pathway inhibition, could cause PIP2 re-localization thereby initiating the events leading, at the peak of MBP expression, to membrane expansion. Importantly, our combined genetic and chemical studies on the role of EGFR in OPC differentiation led to the observation that Gefitinib and Clobetasol combinatorial treatment potently increases MBP expression and OL engagement over single compound activity. This observation opens the way to study this drug combination in RRMS or other demyelination animal models and to their test in combinatorial therapies for their combinatorial remyelination properties.

## Supplementary Materials

The following are available online at https://www.mdpi.com/2073-4409/8/8/844/s1.
