# Supplementary material for: EGFR/ErbB Inhibition Promotes OPC Maturation up to Axon Engagement by Co-Regulating PIP2 and MBP
Source: Cells. 2019 Aug 6;8(8):844. doi: 10.3390/cells8080844 (PMC6721729; doi:10.3390/cells8080844)
Supplement: Supplementary file 1 [file cells-08-00844-s001.zip › Supplementary/Table S2.docx]

**Table S2.**

**Primers pairs for qPCR reactions using SYBR Green master mix.**

| **PRIMERS** | | **FORWARD** | | | | **REVERSE** | | | |
| --- | --- | --- | --- | --- | --- | --- | --- | --- | --- |
| ***GAPDH*** | | 5’-GCTCACTGGCATGGCCTTCCG-3’ | | | | 5’-TGGAAGAGTGGGAGTTGCTGT-3’ | | | |
| ***MBP*** | | 5’-TACCCTGGCTAAAGCAGAGC-3’ | | | | 5’-GAGGTGGTGTTCGAGGTGTC-3’ | | | |
| ***RXRγ*** | | 5’-AGGCAGGTTTGCCAAGCTTCTG-3’ | | | | 5’-GGAGTGTCTCCAATGAGCTTGA-3’ | | | |
| ***NR3C1*** | | 5’-CAAGGGTCTGGAGAGGACAA-3’ | | | | 5’-TACAGCTTCCACACGTCAGC-3’ | | | |
| ***GLI1*** | | 5’-GCTGTCGGAAGTCCTATT-3’ | | | | 5’-ACTGGCATTGCTAAAG-3’ | | | |
| ***GLI2*** | | 5’-CAACGCCTACTCTCCCAGAC-3’ | | | | 5’-GAGCCTTGATGTACTGTACCAC-3’ | | | |
| ***EGFR*** | | 5’- CCAAGAACACACACACCTTTGAG-3’ | | | | 5’- GATGATCTGCAGGTTCTCCAAAG-3’ | | | |
| **esiRNA** | |  | | | |  | | | |
| **Catalogue** | **ID** | | **ID (Ensembl)** | **ID (RefSeq)** | **Gene name** | | **Organism** | **Amount(µg)** | **Concentration** |
| **EMU004971-20UG** | **MU-00497-1** | | **ENSMUSG0000015843** | **NM_001159731**  **NM_009107** | **RXRγ** | | **Mus musculus** | **20.00** | **200 ng/µL** |
| **EMU075311-20UG** | **MU-07531-1** | | **ENSMUSG00000020122** | **NM_007912**  **NM_207655** | **EGFR** | | **Mus musculus** | **20.00** | **200 ng/µL** |
