# Supplementary figures and images for: EGFR/ErbB Inhibition Promotes OPC Maturation up to Axon Engagement by Co-Regulating PIP2 and MBP
Source: Cells. 2019 Aug 6;8(8):844. doi: 10.3390/cells8080844 (PMC6721729; doi:10.3390/cells8080844)

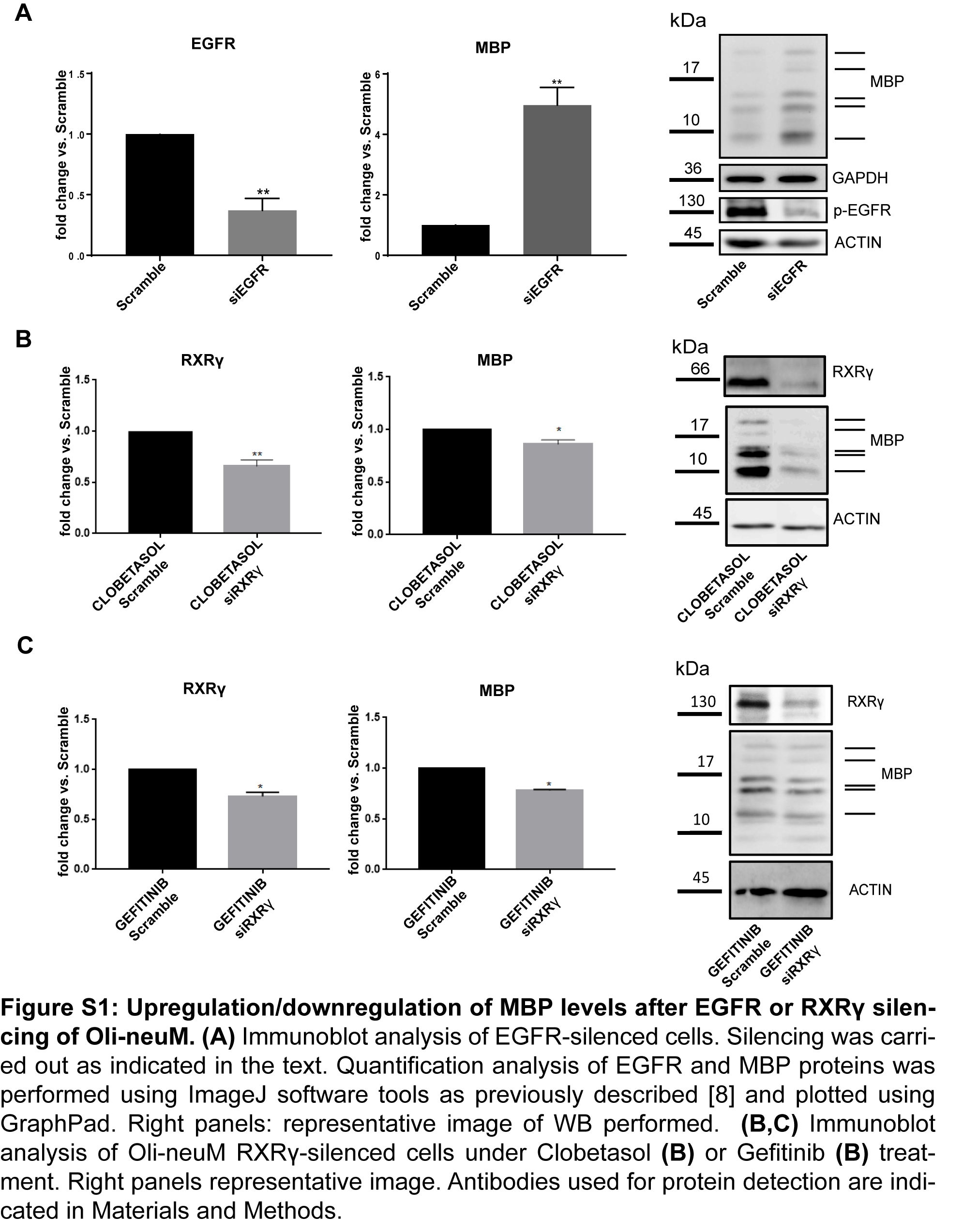

Supplement: Supplementary file 1 [file cells-08-00844-s001.zip › Supplementary/Figure S1.tif]

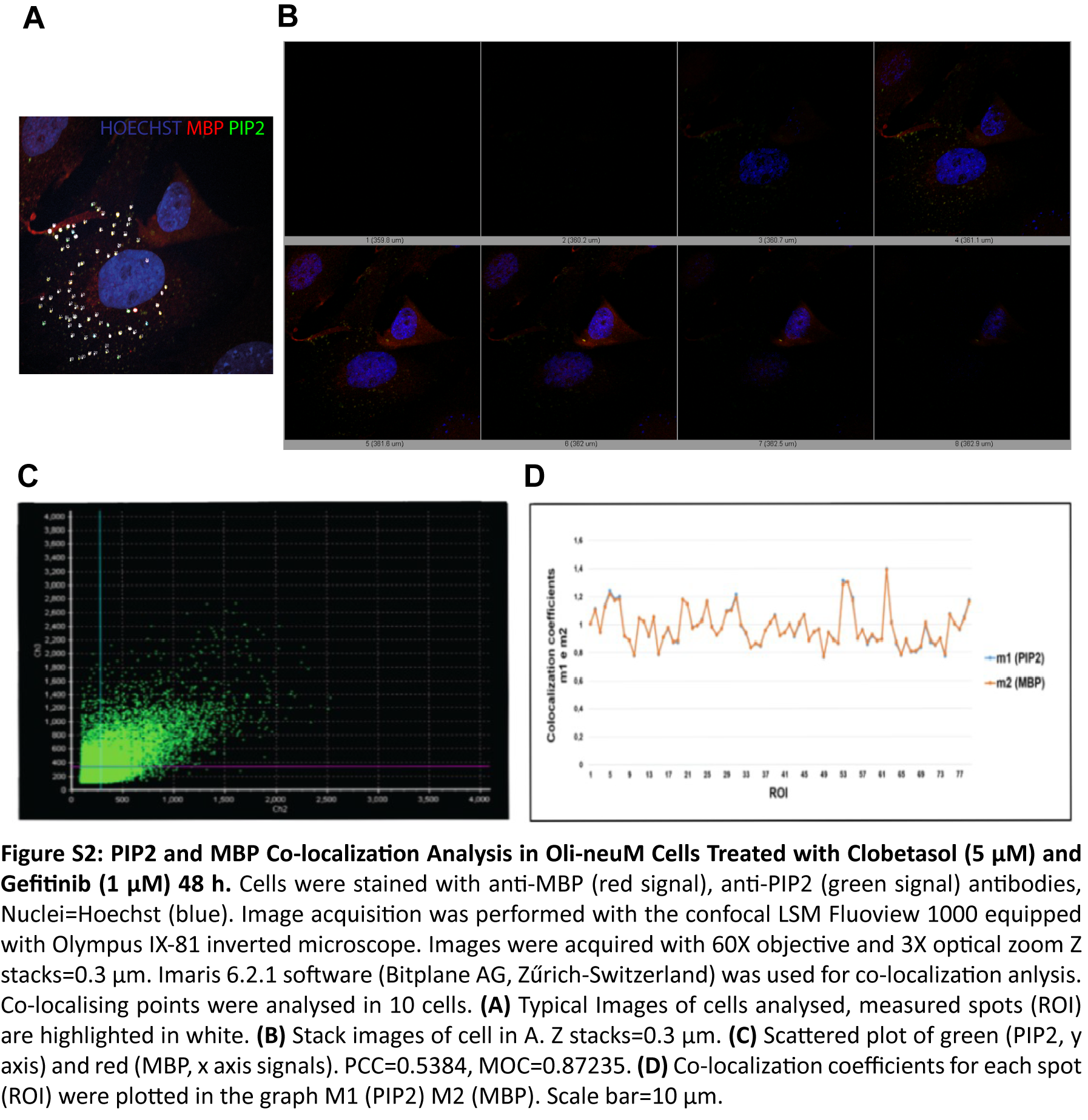

Supplement: Supplementary file 1 [file cells-08-00844-s001.zip › Supplementary/Figure S2.tif]

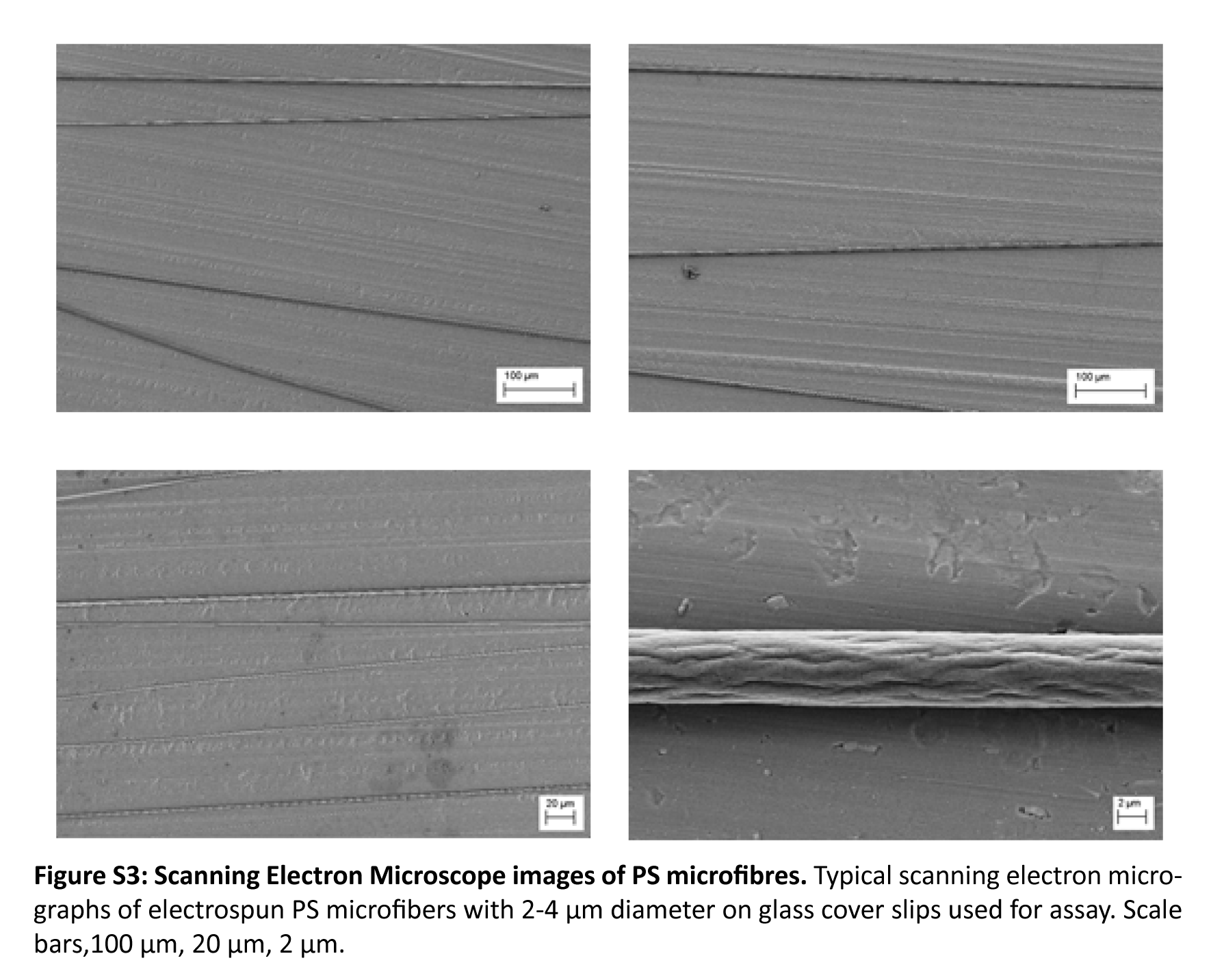

Supplement: Supplementary file 1 [file cells-08-00844-s001.zip › Supplementary/Figure S3.tif]
